# Absolute Quantification of Human Liver Phosphorus-Containing Metabolites *In Vivo* Using an Inhomogeneous Spoiling Magnetic Field Gradient

**DOI:** 10.1371/journal.pone.0143239

**Published:** 2015-12-03

**Authors:** Adil Bashir, Robert Gropler, Joseph Ackerman

**Affiliations:** 1 Mallinckrodt Institute of Radiology, Washington University School of Medicine, St. Louis, Missouri, United States of America; 2 Department of Chemistry, Washington University, St. Louis, Missouri, United States of America; Mayo Clinic, UNITED STATES

## Abstract

**Purpose:**

Absolute concentrations of high-energy phosphorus (^31^P) metabolites in liver provide more important insight into physiologic status of liver disease compared to resonance integral ratios. A simple method for measuring absolute concentrations of ^31^P metabolites in human liver is described. The approach uses surface spoiling inhomogeneous magnetic field gradient to select signal from liver tissue. The technique avoids issues caused by respiratory motion, chemical shift dispersion associated with linear magnetic field gradients, and increased tissue heat deposition due to radiofrequency absorption, especially at high field strength.

**Methods:**

A method to localize signal from liver was demonstrated using superficial and highly non-uniform magnetic field gradients, which eliminate signal(s) from surface tissue(s) located between the liver and RF coil. A double standard method was implemented to determine absolute ^31^P metabolite concentrations *in vivo*. 8 healthy individuals were examined in a 3 T MR scanner.

**Results:**

Concentrations of metabolites measured in eight healthy individuals are: γ-adenosine triphosphate (ATP) = 2.44 ± 0.21 (mean ± sd) mmol/l of wet tissue volume, α-ATP = 3.2 ± 0.63 mmol/l, β-ATP = 2.98 ± 0.45 mmol/l, inorganic phosphates (Pi) = 1.87 ± 0.25 mmol/l, phosphodiesters (PDE) = 10.62 ± 2.20 mmol/l and phosphomonoesters (PME) = 2.12 ± 0.51 mmol/l. All are in good agreement with literature values.

**Conclusions:**

The technique offers robust and fast means to localize signal from liver tissue, allows absolute metabolite concentration determination, and avoids problems associated with constant field gradient (linear field variation) localization methods.

## Introduction

Liver plays a significant role in intermediary metabolism and is an important organ in the study of metabolic diseases. ^31^P magnetic resonance spectroscopy (MRS) has proven to be a useful clinical and diagnostic tool to measure hepatic high-energy phosphates and other phosphorus-containing compounds *in vivo* (hereafter, “phosphorus metabolites”). Alterations in phosphorus metabolites have been seen in various diseases such as cirrhosis [1–3], non-alcoholic fatty liver disease [4, 5], viral hepatitis [6, 7] and diabetes mellitus [8–10]. Levels of these ^31^P MRS detected phosphorus metabolites can be quantified as relative metabolite resonance-amplitude ratios (hereafter, “metabolite ratios”) or as absolute concentrations. Absolute quantification is desirable since metabolite ratios are susceptible to complications due to simultaneous changes in metabolite content with disease.

Several studies reporting absolute quantification of phosphorus metabolites in human liver have been previously published [11–16]. Most commonly used techniques for ^31^P signal localization and absolute quantification are chemical shift imaging (CSI) [17] and image selected *in vivo* spectroscopy (ISIS) [18]. CSI has the advantage of providing spatial information but suffers from long data acquisition time and is susceptible to motion induced artefacts [11, 14]. ISIS is a well-established technique for ^31^P MRS localization and several studies have used it for quantification of phosphorus metabolites in human liver [12, 16]. However, ISIS is an eight-scan acquisition scheme and is rather susceptible to motion artifacts, since movement can lead to incomplete cancellation of the large amount of unwanted signal from outer volume. Adiabatic pulses are generally used with ISIS sequence to achieve uniform RF excitation and reduce chemical shift displacement error (CSDE). CSDE error is generally minimized by increasing the RF bandwidth by using a short duration RF pulse. Available RF power, maximum gradient field amplitude and increased power deposition can limit the approach when used at high field magnets.

Crowley and Ackerman [19], and Chen and Ackerman [20, 21] described the use of a superficial and highly inhomogeneous magnetic field gradient to eliminate the signal contribution arising from surface tissue in rodent liver ^31^P MRS. This approach offers simple and fast localization of the MRS signal from liver and is independent of the limitations that accompany methods based on constant field gradients and frequency selective pulses. This approach has been demonstrated in a limited number of human studies for different applications [22–24]. Jehensen and Bloch [25] demonstrated the feasibility of this surface-spoiling technique for human liver studies however, no attempt was made to determine phosphorus metabolite ratios or to calculate absolute molar concentrations. The ^31^P MRS rat liver study by De Bisschop *et al*. [26] is the only report employing surface spoiling that derived absolute phosphorus metabolite concentrations.

The aim of the research reported herein was (i) to set up a robust and fast method to localize ^31^P MRS signals from *human* liver using a surface spoiling inhomogeneous magnetic field gradient, and (ii) to measure absolute molar concentrations of phosphorus metabolites in *human* liver on a 3-T clinical scanner. To accomplish these goals, a local spoiling gradient coil was constructed to achieve spoiling depths expected to be required in human studies. The performance of the apparatus and technique was experimentally verified using a two-compartment phantom. The device was then employed for quantitative ^31^P MRS studies of human liver.

## Theory

Detailed theory can be found in previous reports [21, 25] here a brief summary describing the technique is presented. The approach is based on local signal-spoiling arising from intra-compartment dephasing of spins, which is achieved by applying a local inhomogeneous magnetic field gradient in the time between signal excitation and data acquisition. The gradient coil should be such that it does not allow the penetration of the inhomogeneous gradient field into the targeted deep-lying tissue from which the signal is desired. The gradient coil design used herein consists of a linear array of antiparallel current elements, each running perpendicular to the main magnetic field. This design is similar to that presented previously for animal studies [20] and has been termed a “meanderline” design in an application to nuclear quadrupole resonance spectroscopy [27].

**Fig 1:**
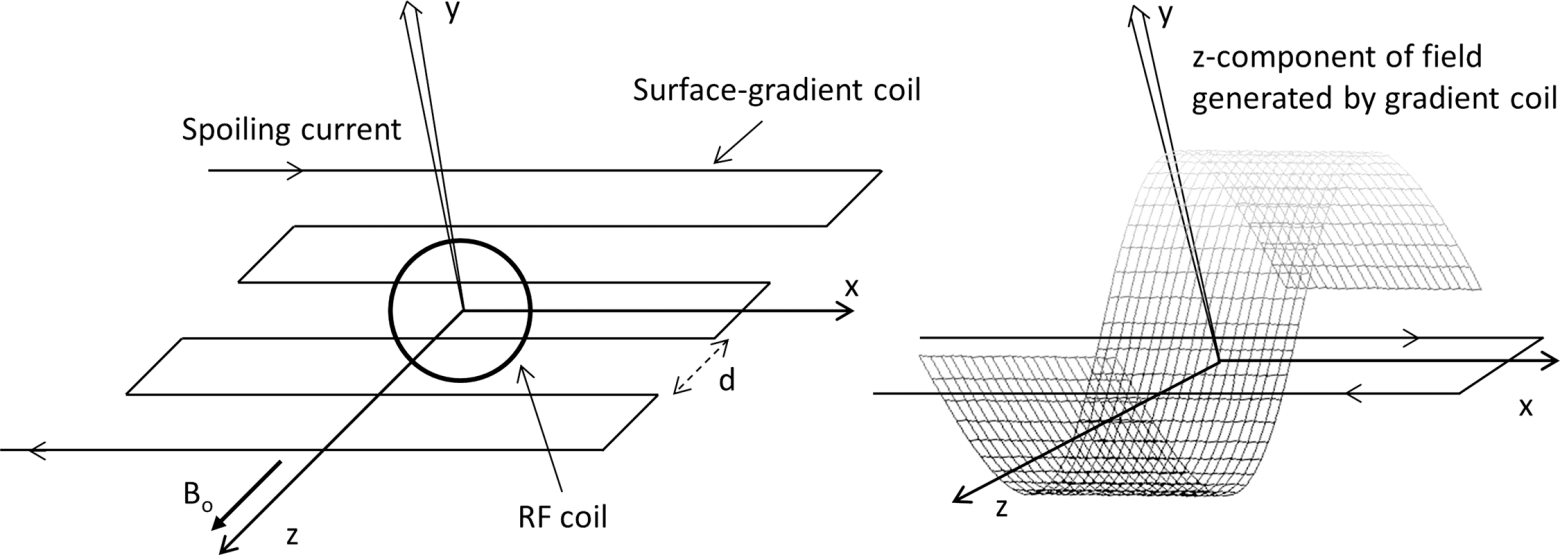
(a) The orientation and geometry of the gradient spoiling coil and the RF coil. The current reverses direction at alternate current segments. This arrangement will generate a magnetic field gradient along the y- and z-axis and the gradient along x-axis will be essentially zero. (b) The representative magnetic field *B*_*z*_(*q*) distribution resulting from the surface spoiling gradient coil. The maximal positive field in a given XZ plane appears directly above one wire, the maximal negative spoiling field appears at the adjacent wire, and the zero spoiling field appears at the midpoint between the two wires.

The layout and orientation of the surface-spoiling gradient coil and RF coil is shown in Fig 1. A current through the gradient coil generates an inhomogeneous magnetic field in the XZ-plane where the specific field strength at any point in the sample depends upon its position, i.e., coordinate *q* (*q* = *x*, *y*, *z*). The imposed inhomogeneous magnetic field induces a local position-dependent phase shift and the signal spoiling arises from dephasing of spins in the inhomogeneous field. Only the Δ*B*(*q*) component parallel to main magnetic field Δ*B*_*z*_(*q*) is relevant for required dephasing (S1 Script). As long as the gradient is not applied during RF excitation pulse(s) and data acquisition, the local phase shift is given by

$$\phi (q)=\gamma \Delta B_{z}^{\prime }(q)\int_{0}^{T}I(t)dt=\gamma \Delta B_{z}^{\prime }(q)Q$$

where γ is the gyromagnetic ratio, $\Delta B_{z}^{\prime }(q)$ is the spoiling magnetic field at coordinate *q* produced by unit current in the spoiling gradient coil, *I* is the current through the coil, *T* is the duration of the gradient pulse, and *Q* is the charge through the gradient coil. Assuming the surface-spoiling gradient coil is large compared to the RF coil and the RF field (B_1_) is homogeneous, the signal originating within a uniform tissue can be expressed as

$$S\propto \int_{y_{i}}^{y_{f}}\int_{-d/2}^{d/2}\exp\left(i\phi (z)\right)dzdy$$

where *y* is the depth coordinate and *y*_*i*_ and *y*_*f*_ are the intial and final depths of the targeted tissue (i.e., targeted for suppression or for detection), and *d* is the distance between the adjacent current elements. Integration is performed only over the region–*d/2* to *d/2* since the gradient field repeats itself due to the meanderline coil geometry. Relaxation effects were not taken into account but in practice the duration of the spoiling gradient pulse is short relative to typical *in vivo* relaxation times. The variation of signal phase over the region experiencing the inhomogeneous field causes signal suppression.

**Fig 2:**
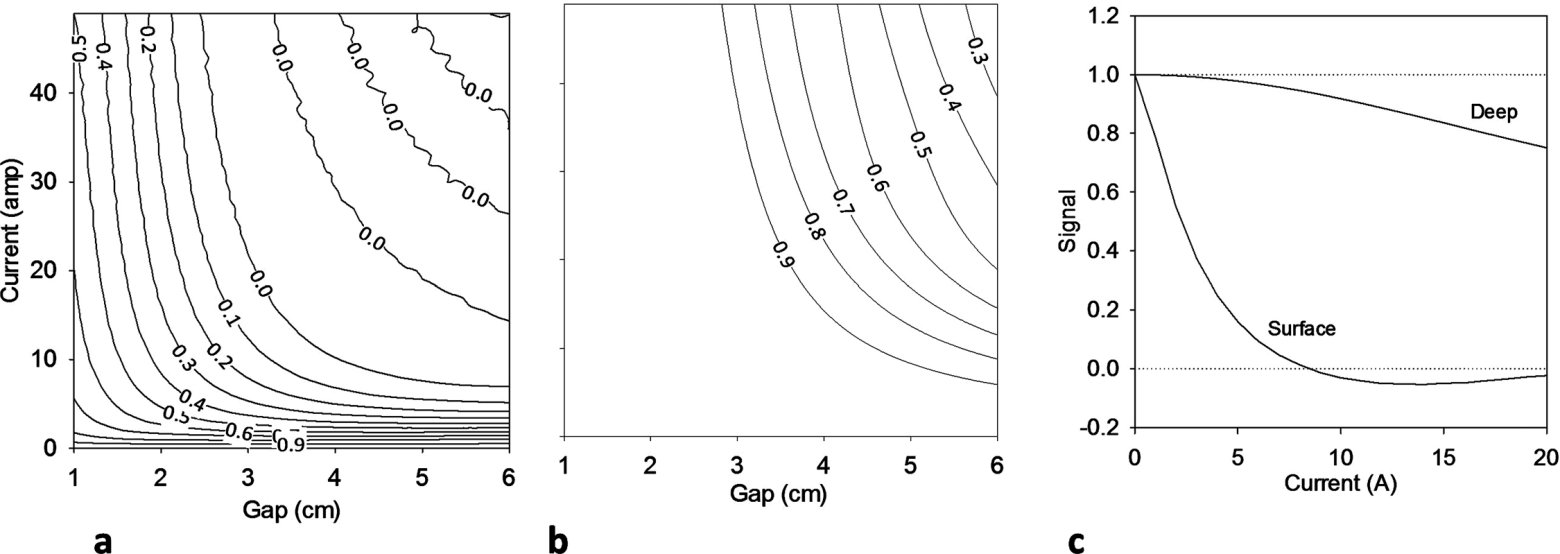
The calculated relative ^31^P signal amplitudes for the two-compartment phantom. Transmission and reception B_1_ was assumed homogeneous over the sample. (a) Relative signal from the surface-lying compartment as function of current through the surface gradient coil and distance between the current elements of the gradient coil. The gradient duration was set to 1 ms for these simulations. The total relative signal quickly decreases to zero as the gap or the current is increased. (b) Relative signal from the deep-lying compartment. The total signal stays above 90% for practical values of current and gap between conductors with relatively small decrease for large current or gap. (c) The calculated relative signal amplitude of the two compartments when the distance between the surface gradient coil elements is fixed at 4.5 cm. The signal from the surface-lying compartment decays markedly as the gradient driving current increases, approaching and staying near 0 for current above 8 amp. The signal from deep compartment decreases more moderately with 8.2% loss of signal for 10 amp current.

A two-compartment computer model representing human chest and liver was used for simulations. The chest was assumed to lie in a region 0.2 cm to 4 cm below the surface gradient coil. The liver was assumed to extend from a region 4 cm to 8 cm below the surface gradient coil with no empty space between the chest and liver. Simulated signal as a function of spacing between the gradient coil current elements and the current through the coil is shown in Fig 2. A gradient duration of 1 ms was chosen for these simulations. As the spacing between current elements or the current through the conductors increases, signal from the slab next to the gradient coil decrease markedly and is reduced to ~5% residual signal, oscillating around zero. Additionally increasing current or gap between conductors does not further affect signal suppression from the surface compartment.

The signal from the deep compartment is seen to decreases slightly with increasing current and gap between conductors, an undesired consequence of the inhomogeneous gradient field penetrating the compartment. Fig 2C shows signal from the two compartments as function of current for a gradient coil spacing of 4.5 cm. As the current is increased, the signal from the surface compartment decreases rapidly and oscillates near zero whereas the signal from the deep compartment decreases relatively slowly. Thus, a current of 8–12 amps is expected to reduce the surface signal by 95% whereas the deep compartment signal is reduced by 4–10%. Based on these simulations, a spacing of 4–5 cm is appropriate for human applications with modest current requirements.

### RF and gradient coil design

**Fig 3:**
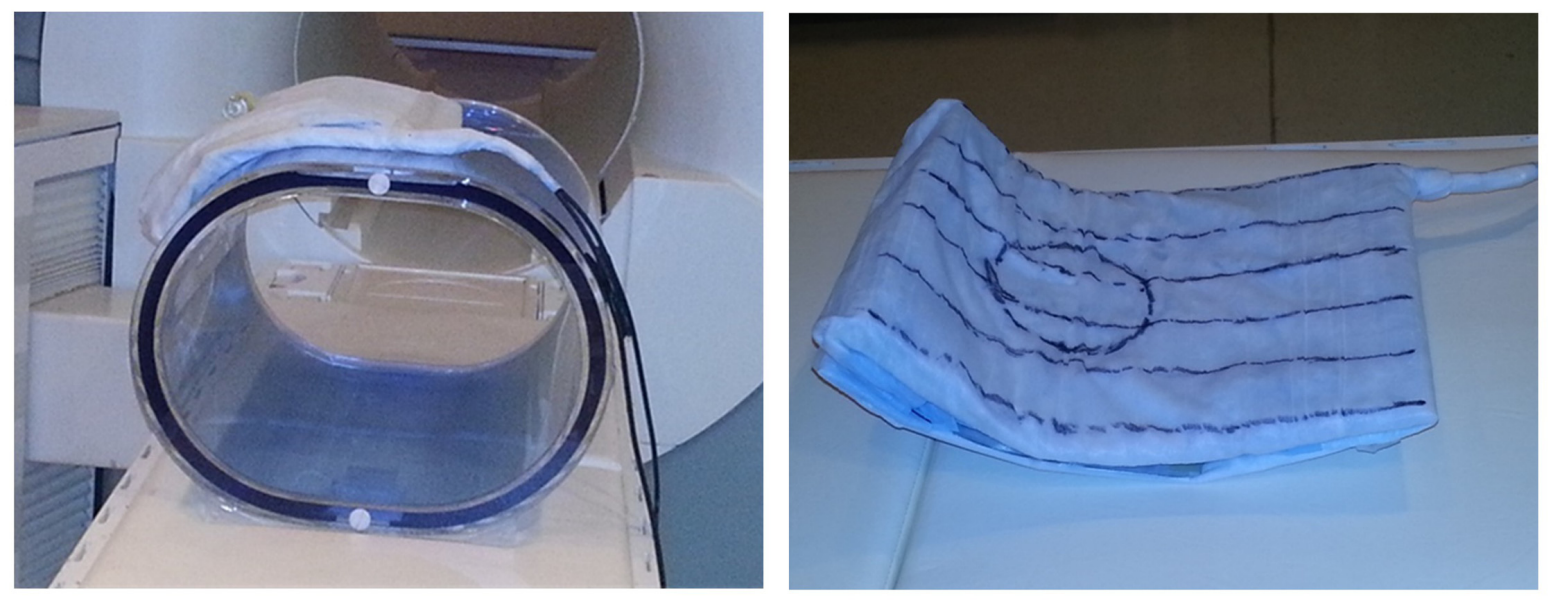
Photograph and layout of surface spoiling gradient coil and RF coil. The gradient/RF coil assembly was bent to contour around the torso.

Experiments were carried out on Siemens 3-T (Siemens, Erlangen) TRIO scanner equipped with broad band capabilities. The gradient coil was built on a solid polycarbonate sheet (25 cm by 30 cm) bent to contour around the human torso with 4.5 cm distance between the current elements. The ^31^P RF coil consisted of an 10-cm single-turn surface coil centered on the gradient coil assembly (Fig 3). Either gradient or RF coil can be placed immediately adjacent to the subject; however, for this demonstration the gradient coil was placed next to the subject and a 1/8 inch polycarbonate sheet separated the gradient and RF coils. Current to the gradient coil was provided by a laboratory constructed MOSFET switch turned on by a TTL pulse. The timing of the gradient switching was controlled by the optical sync pulse from the scanner console and an optical to TTL converter was used to drive the MOSFET. The current amplitude was controlled by a variable resistance in series with the gradient coil. A 10-MHz low pass filter eliminated potential sources of noise from the driver circuit.

## Methods

The study was reviewed and approved by Human Research Protection Office at Washington University in St. Louis. A written informed consent was obtained from all participants before the study.

### Spoiling-Spoiling Gradient Coil Validation

#### Two-compartment phantom validation

**Fig 4:**
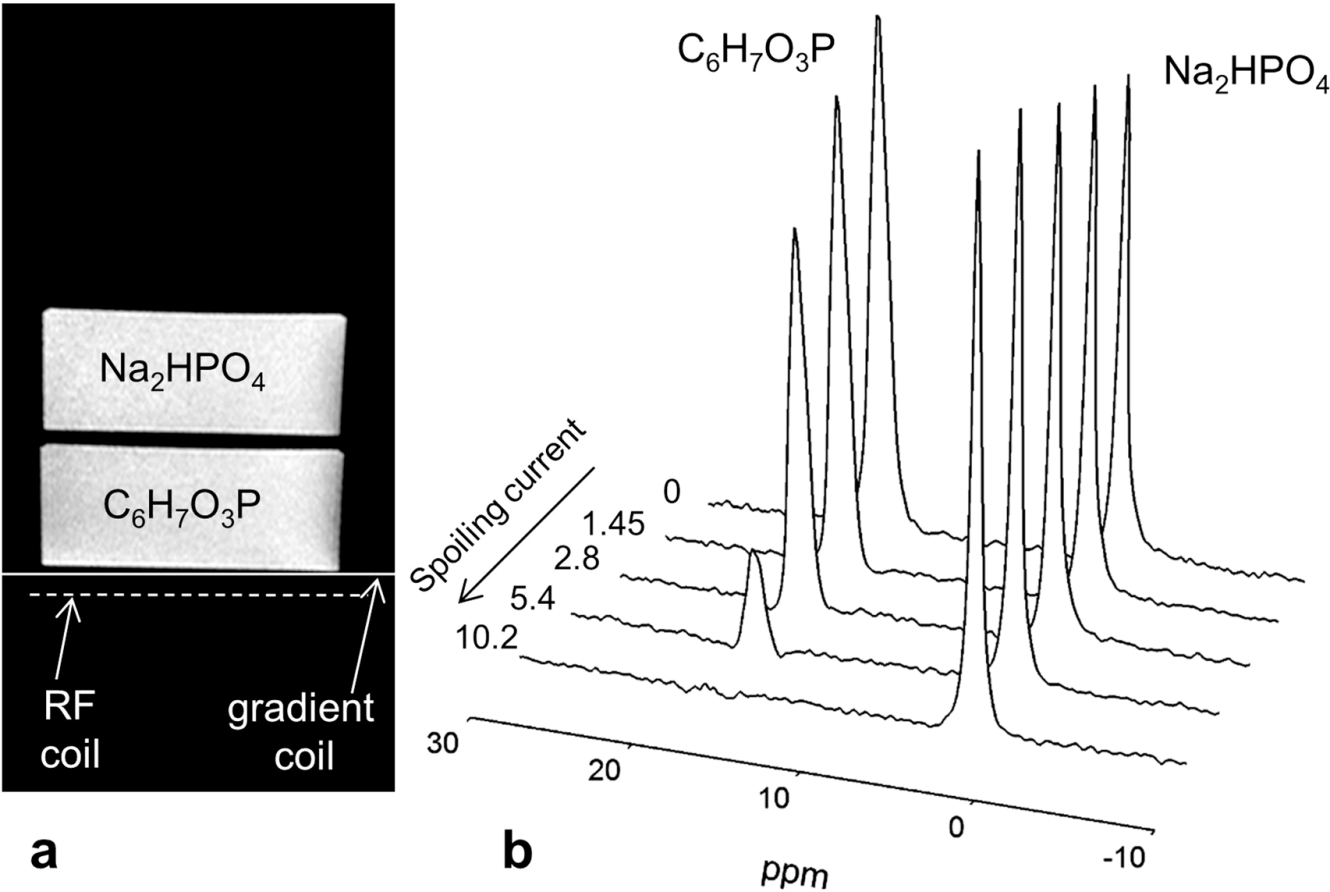
(a) Experimental arrangement of the two-compartment phantom, surface spoiling gradient and RF coils. The height of each compartment is 4 cm. (b) Series of spectra from the two-compartment phantom as the current through the gradient coil is increased. The signal from the surface-lying compartment (bottom) decreases quickly and disappears essentially completely. The current through the gradient coil is displayed with each spectrum. Spoiling gradient duration is 1 ms.

Optimal spoiling gradient current was initially determined in a two-compartment phantom consisting of 3.6-cm deep plastic disks containing 150-mM phenylphosphonic acid (C_6_H_7_O_3_P) and 150-mM sodium phosphate (Na_2_HPO_4_), both giving distinct ^31^P resonances at 18.5 and 3.3 ppm, respectively, relative to phosphocreatine which is assigned 0.0 ppm, vide infra (Fig 4, Table A in S1 File). A pulse and acquire sequence was used with the data acquisition delayed to accommodate the 1 ms spoiling gradient pulse. Data acquisition was started 0.1 ms after the gradient pulse was turned off to ensure that the gradient field had decayed to zero before acquisition. Current through the spoiling gradient coil was varied from 0–10.2 amps. The following acquisition parameters were used for the two-compartment phantom experiment: 2.56 ms hyperbolic secant (HS) adiabatic excitation pulse applied at a center frequency midway between C_6_H_7_O_3_P and Na_2_HPO_4_ resonances, TR = 2 s, averages = 8, spectral width = 3000 Hz, and 512 data points.

#### RF pulse calibration

**Fig 5:**
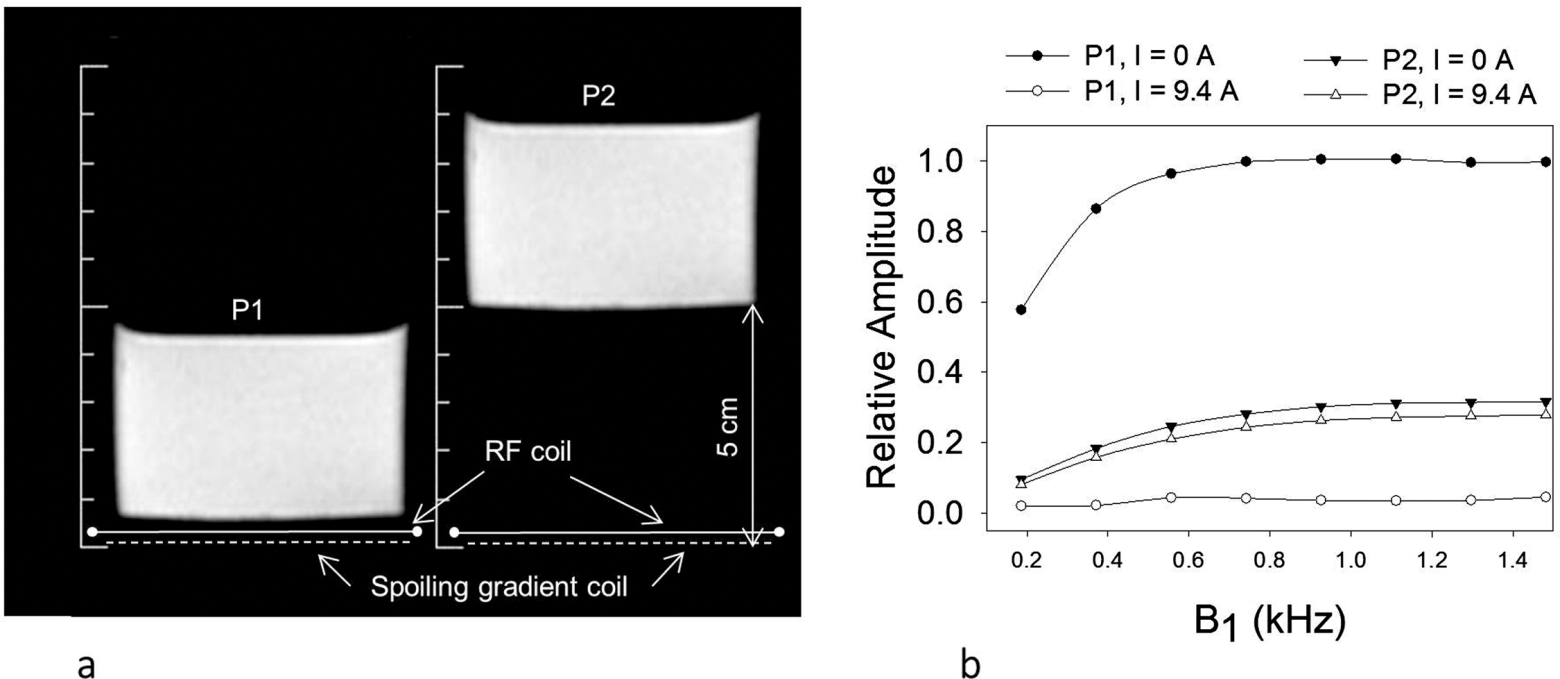
(a) Images of phantom showing its location relative to the gradient (solid line) and RF coil (dashed line). (b) The circles show the signal from phantom placed next to the coil assembly (left) and triangles the signal when the phantom was placed 5 cm from the coil assembly. Solid symbols show the signal intensity with 0 amps and open symbols show 9.4 amps driving current through the gradient coil. B_1_ field of 1.4 kHz ensured that the RF excitation pulse was adiabatic for the deep lying phantom. A small decrease in the signal from the deep lying phantom was observed (open vs closed triangles) due to inhomogeneous field penetrating the deep phantom. No signal was detected from the surface phantom with 9.4 amp driving current for all B_1_ field strengths.

The RF pulse efficacy in absence and presence of inhomogeneous spoiling gradient coil was calibrated using 3.8-cm deep and 6-cm diameter phantom containing 150-mM Na_2_HPO_4_ (Fig 5, Table B in S1 File). For the first series of experiments the phantom was placed directly next to the gradient/RF coil assembly mimicking surface tissue. Data was acquired using an on resonance 2.56 ms HS excitation pulse centered at Na_2_HPO_4_ resonance frequency and B_1_ field was varied from 0.2 kHz to 1.5 kHz and a driving current of 0 amps and 9.4 amps for the surface-spoiling gradient coil. Other acquisition parameters were same as given above. For the second series of experiments the phantom was placed approximately 5 cm from the gradient/RF coil assembly and the experiments were repeated.

The efficiency of an adiabatic excitation pulse degrades as the resonance offset increases. The off-resonance efficiency of the adiabatic half passage excitation pulse employed herein was determined by varying the offset frequency from -12 ppm to 12 ppm with an increment of 2 ppm with the point phantom placed 5 cm from the gradient/RF coil assembly. Excitation efficiency was calculated as the resonance amplitude of Na_2_HPO_4_ at a given offset frequency normalized to that of the resonance from Na_2_HPO_4_ at offset = 0.

#### *In vivo* human validation

*In vivo* performance of the spoiling gradient coil was tested with two adult subjects who consented to a protocol approved by local ethics board. The subjects were placed supine and the RF and gradient coil assembly was placed directly over the chest. The gradient coil was wrapped partially around the side of the subject’s body. ^1^H MR scout images were taken using a gradient recalled echo (GRE) pulse sequence with the body coil to confirm the correct positioning of the ^31^P RF coil and to perform automatic shimming. The image acquisition parameters were: repetition time (TR)/echo time (TE) = 10/2.76 ms, slice thickness = 10 cm, field of view = 45 x 45 cm^2^, matrix size = 512 x 512 with 4 averages. ^31^P spectra from liver were obtained using a pulse and acquire sequence employing a 2.56 ms adiabatic half passage excitation pulse centered at PCr resonance frequency followed by 1 ms delay to accommodate the gradient pulse. Data acquisition began 0.1 ms after turning off the gradient pulse with effective TE = 1.1 ms. Other sequence parameters are TR = 2 s, 96 averages, spectral width = 3000 Hz, and 512 data points.

### *In Vivo* Human Liver High Energy Metabolite Quantification

#### Human subjects

Eight healthy adults were recruited for the study and provided informed written consent. The average age of the subjects was 30.5 ± 11.1 years (range 23–55 years) and body mass index (BMI) of 24.6 ± 3.5 kg/m^2^ (range 20.3–29.5 kg/m^2^). All studies were done non-fasting and approximately same time (between 3pm and 5pm) in the afternoon.

#### ^31^P MRS of Human liver

Subjects were placed supine with surface gradient and RF coils over the torso. A vial (2.5 cm diameter and 5 cm long) containing 150 mM C_6_H_7_O_3_P was fixed to the center and approximately 4 cm from the plane of RF coil. The spectra form subjects and reference phantom were compared to the signal from this vial to account for changes in coil efficiency and loading. This approach has been previously validated and described in detail [26]. ^1^H MR scout images were acquired, *vide supra*, and ^31^P spectra from liver were obtained using a pulse and acquire sequence employing a 2.56 ms adiabatic half passage excitation pulse centered at PCr resonance frequency followed by 1 ms delay to accommodate the gradient pulse. A driving current of 9.4 amps was used for the surface-spoiling gradient coil as determined from the coil validation experiments. Other sequence parameters are TR = 3 s, 128 averages, spectral width = 2500 Hz, and 1024 data points. A second spectrum was also acquired by moving the excitation pulse frequency to be on resonance with C_6_H_7_O_3_P and the current through the surface gradient coil turned off. All acquisition parameters were the same except number of averages = 32.

#### ^31^P MRS phantom

**Fig 6:**
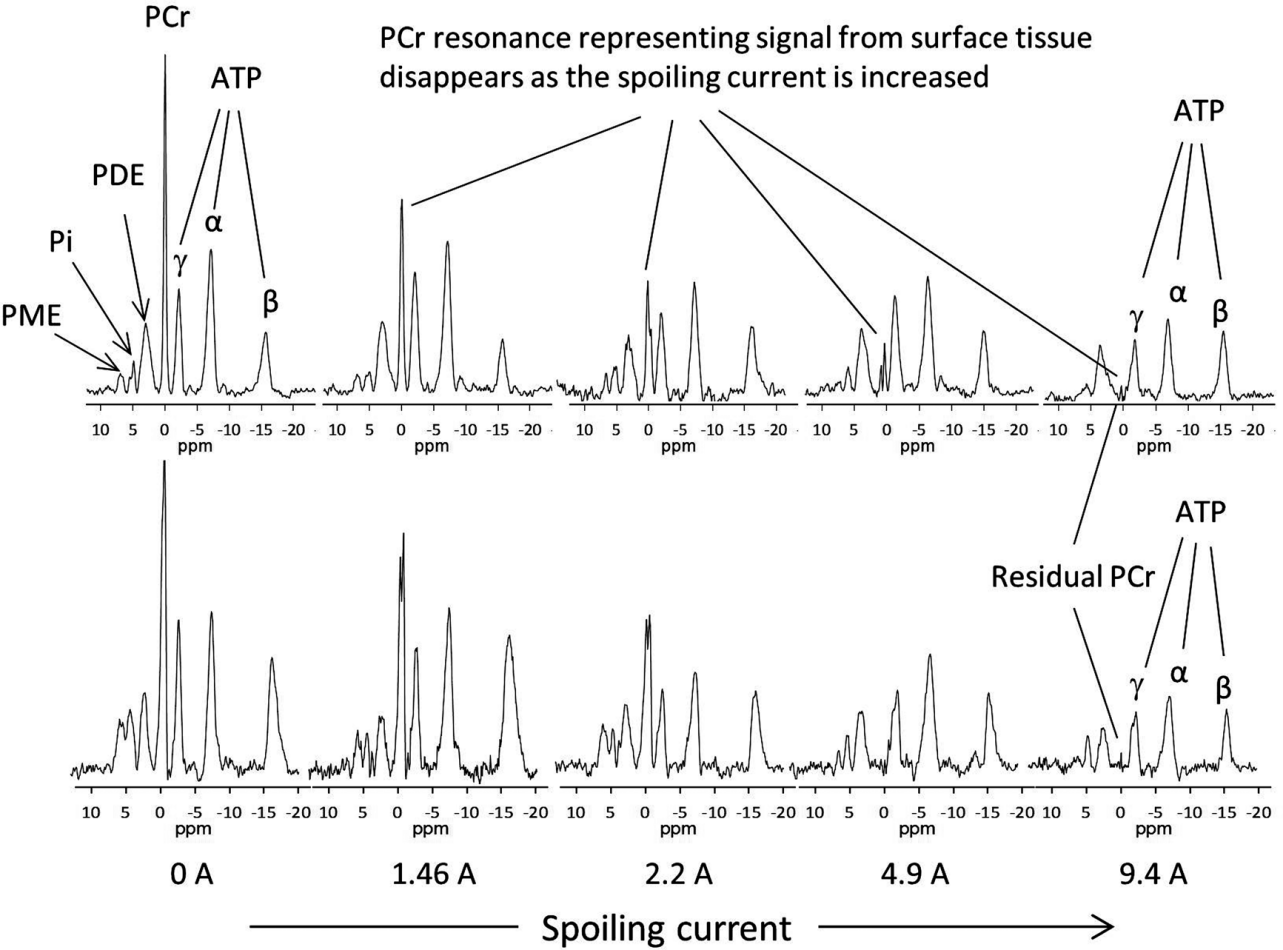
^31^P MR spectra from *in vivo* liver (two subjects) with varying surface gradient current at a fixed gradient duration of 1 ms. PCr exists in muscle tissue but not in liver and its signal provides a convenient marker for the contribution (contamination) of muscle tissue to the detected spectrum. Each spectrum is a result of 92 scans at 2-s repetition time for a total acquisition time of 3.2 minutes. The spoiling gradient driving current (A, amps) is given below the respective spectrum. The PCr signal quickly decreases as the current is increased and for both subjects the resonance is eliminated for driving current of 9.4 A. Abbreviations are defined in the main text.

Measurement aimed for absolute quantification requires that the volume contributing to the signals and the excitation geometry is the same in the phantom and animal experiments. For calibration, a cylindrical phantom consisting of 52 mM/L Na_2_HPO_4_ cylindrical vessel with a total volume of 4 L. The sensitivity of the RF coil was determined using MATCOIL software assuming uniform excitation as shown in Fig 6 (Table C in S1 File). Signal localization was further tested by acquiring a 1D profile of the phantom in a plane perpendicular to the surface of the coil, i.e., signal was detected in the presence of readout gradient perpendicular to the coil surface. Acquisition parameters were as follows: field of view = 40 cm, TR = 2 sec, TE = 1.1 ms, matrix size = 64 and signal averages = 64. 1D profiles were acquired with driving currents of 0 amps and 9.4 amps for the surface-spoiling gradient coil.

For absolute concentration calculations, ^31^P MRS data were acquired using a pulse and acquire sequence with a surface-gradient driving current of 9.4 amp, TR = 50 s, and 8 averages. Reference data were acquired by moving the excitation frequency to that of the C_6_H_7_O_3_P acid resonance and data were acquired with the current through the surface gradient turned off, TR = 3 s and 32 averages.

### Data Analysis

#### Spectral processing

We used six resonances to model *in vivo* spectra. Phosphoethanolamine (PE) and phosphocholine (PC) were represented as phosphomonoester (PME) resonance at 6.6 ppm, glycerophosphoethanolamine (GPE) and glycerophosphocholine (GPC) were represented phosphodiester (PDE) resonance at 3.2 ppm. The other four resonances consisted of inorganic phosphate (Pi) at 5.2 ppm, and the γ-, α-, and β-phosphates of ATP (γ-, α-, and β-ATP) at -2.5, -7.6 and -16 ppm, respectively. These ^31^P chemical shift assignments are relative to the ^31^P resonance from phosphocreatine (PCr), when present, being assigned 0.0 ppm. For quantification purposes, all data were processed in the time domain by the AMARES (advanced method for accurate, robust, and efficient spectral fitting) algorithm implemented in the jMRUI software package [28, 29]. No phase correction or line broadening was applied before spectral fitting. *A priori* knowledge consisted of soft constraints on the resonance frequency. Truncation of the first time-domain data point reduced the signal contribution from rapidly decaying background signals (e.g., semi-solid-like membrane bound phosphorus containing moieties). For display purposes, following application of a 15 Hz line broadening exponential apodization function, time-domain data were Fourier transformed and the resulting frequency-domain spectra were manually phase corrected.

### Absolute Metabolite Concentration Calculations

The double standard method was implemented as described previously [26]. Measured off-resonance excitation efficiency was used to correct the resonance amplitudes of PME, Pi, PDE, and ATP obtained from *in vivo* experiments. The amplitudes of the *in vivo* metabolite resonances were corrected for partial saturation using the reported T_1_ relaxation times of the liver phosphorus metabolites at 3 T [30].

Assuming that the volume of the excitation is the same for phantom and *in vivo* experiments the concentration of phosphorus metabolites *in vivo* can be calculated as

$$c_{m}^{i}=\frac{A_{m}^{i}}{A_{p}}∙\frac{A_{r}}{A_{r}^{\prime }}∙c_{p}∙s_{m}^{i}$$

where $c_{m}^{i}$ is the molar concentration of the *i* ^*th*^ metabolite, $A_{m}^{i}$ is the amplitude of the *i* ^*th*^ resonance obtained *in vivo*, *A*_*p*_ is the resonance amplitude of the sodium phosphate phantom, *A*_*r*_ is the resonance amplitude of the C_6_H_7_O_3_P reference attached to the coil in the phantom experiment, $A_{r}^{\prime }$ is the resonance amplitude of the C_6_H_7_O_3_P reference obtained in the human experiment, *c*_*p*_ is the concentration of the phantom solution, and $s_{m}^{\prime }$ is the correction factor for off-resonance excitation efficiency of the adiabatic pulse.

## Results

### Two-Compartment Phantom Validation

The experimental configuration for the phantom experiments is shown in Fig 4A with the bottom (deep-lying) compartment of the phantom containing C_6_H_7_O_3_P. A series of spectra, Fig 4B, show increasing efficiency of the surface-spoiling gradient coil as the gradient strength (current) is increased. Spins from the surface compartment lose phase coherence and the signal disappears almost completely with a driving current of 9.4 A and a duration of 1 ms. Signal from the deep-lying compartment clearly remains, decreasing only by 4.1% with a gradient driving current of 9.4 A. With a significantly larger driving current there was unwanted penetration of gradient field in the deeper regions as seen with a reduction of the sodium phosphate resonance amplitude.

### RF pulse calibration

When the spoiling-gradient driving current was 0 amps the signal from the surface phantom increased with increasing B_1_ and came to steady state for B_1_ = 0.8 kHz in absence of inhomogeneous gradient field (Fig 5). The signal was near zero for all B_1_ when the spoiling-gradient driving current in the gradient coil was 9.4 amps. The signal from deep phantom increased with increasing B_1_ for both 0 amps and 9.4 amps and reached steady sate at a B_1_ field strength of approximately 1.1 kHz. B_1_ field strength of 1.4 kHz was employed for all *in vivo* and reference phantom experiments thus ensuring that the pulse was adiabatic over most of the sensitive region of the coil.

### *In Vivo* Human Validation

A series of spectra from the liver of these two initial test subjects is shown in Fig 6. As expected, when there is no current through the spoiling-gradient coil a PCr signal is present, indicating the presence of signal contribution (“contamination”) from the superficial muscle tissue. Resonance intensities decrease as the current through the spoiling-gradient coil is increased. The PCr signal intensity completely disappears to within the noise when the driving current is 9.4 A.

### *In Vivo* Quantification

**Fig 7:**
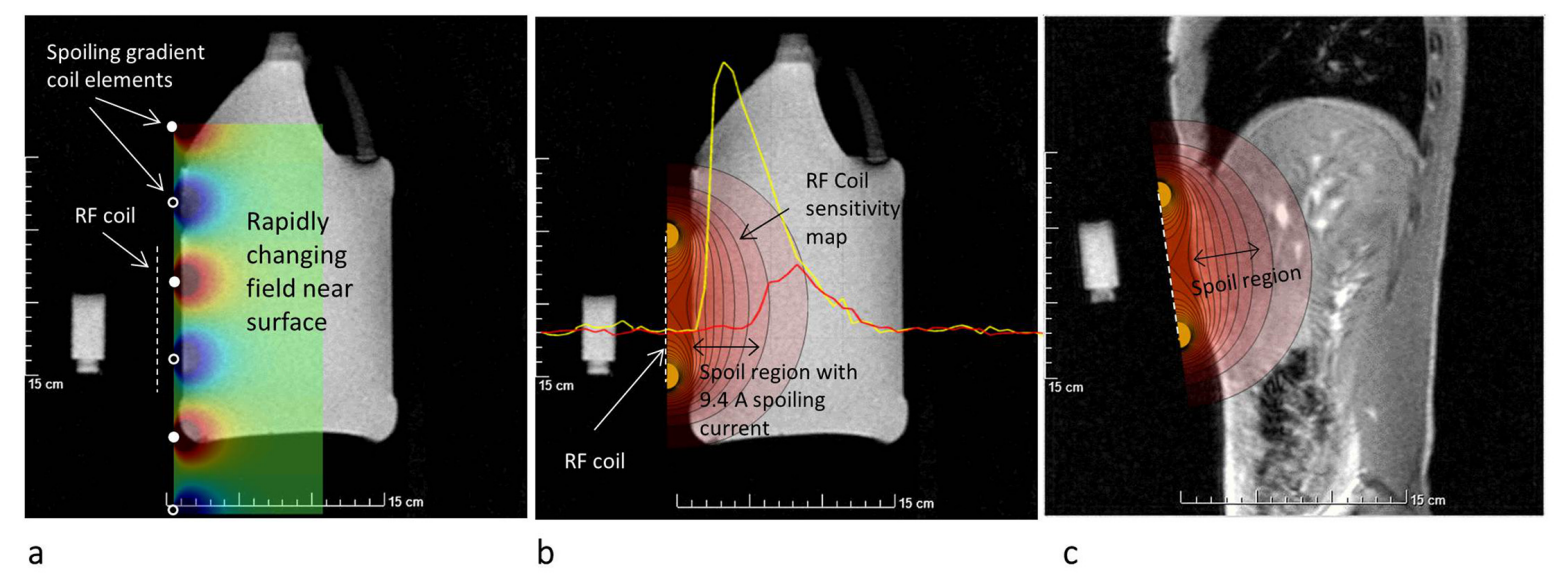
(a) Image of phantom and layout of surface spoiling and RF coil. The inhomogeneous magnetic field is localized near the surface. (b) Coil sensitivity map and 1D profiles displayed over the phantom image. Profile in absence of spoiling gradient (yellow) shows high signal intensity near the surface. The red curve shows the signal when 9.4 amps was used as the driving current in the gradient coil. The signal is confined to the deep region. (c) Coil sensitivity map displayed over the *in vivo* image.

Fig 7 shows localizer images of phantom, and through the liver and layout of the spoiling-gradient and RF coils. The C_6_H_7_O_3_P phantom marking the center of the RF coil is visible in the images. The elements of the spoiling-gradient coil ran perpendicular to the plane of the images. The schematic overlay shows the gradient field generated a rapidly changing field near the surface (Fig 7A). The figure also shows the coil sensitivity plots overlaid over the phantom and *in vivo* localizer image. The plot shows the 1D profiles in absence and presence of surface spoiling gradient field (Fig 7B Table D in S1 File). The profile in presence of gradient field shows that the signal is expected to be localized to the liver region.

**Fig 8:**
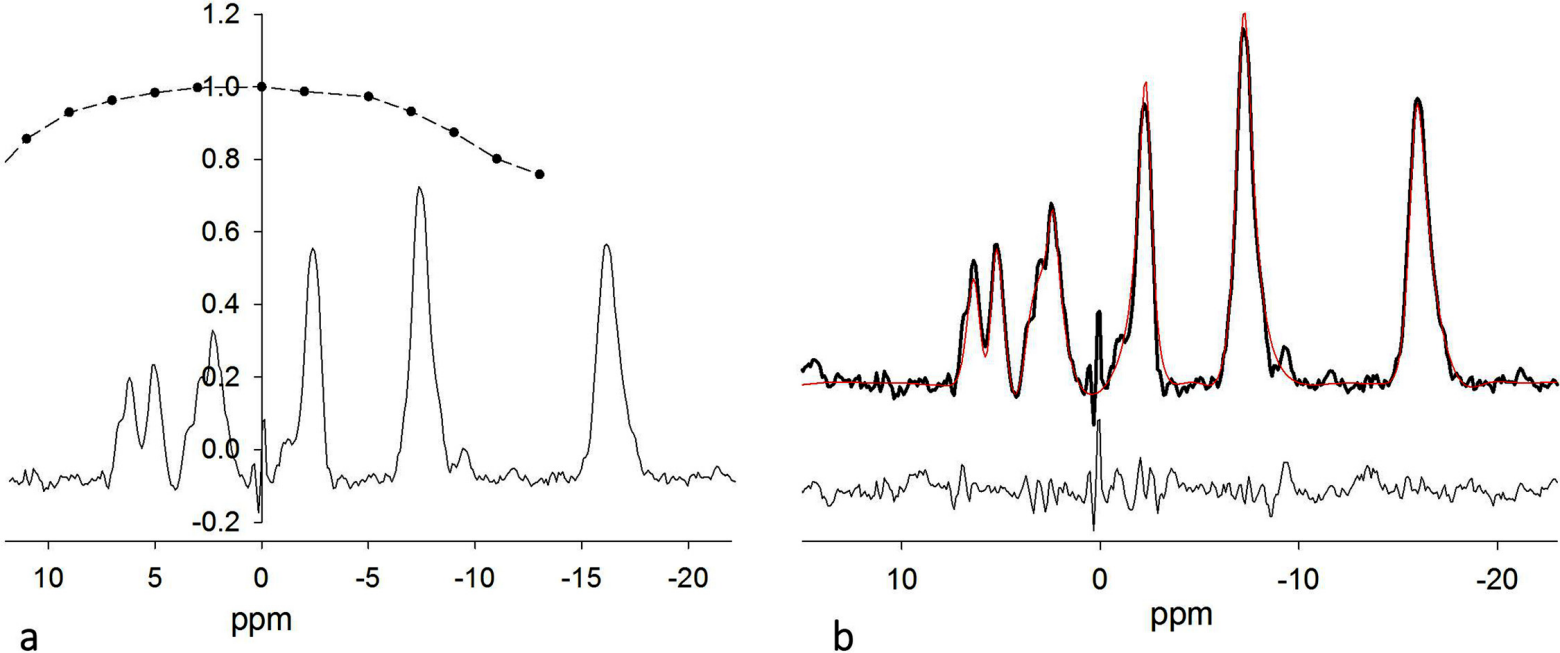
(a) ^31^P spectra from *in vivo* liver and the curve representing the off-resonance efficiency of the adiabatic excitation pulse. (b) Example of the six-resonance model AMARES quantification. The model (shown in red) is superimposed over the original data set. The residual spectrum is shown below.

The excitation pulse had adequate bandwidth to excite all the relevant resonances (Fig 8A Table E in S1 File). A correction factor of 4% was needed for PME resonance. The correction factors for Pi, PDE and γ-ATP were less than 2% and were considered within the noise and no correction was applied. The pulse efficiency dropped quickly towards the α- and β-ATP resonances, and correction factor 0f 0.94 and 0.75 were used to correct the measured resonance amplitude. Fig 8B shows an example of AMARES quantification with Fourier transformation of the estimated six-resonance time-domain model superimposed over the original data set. The low intensity of the PCr signal confirmed excellent suppression of the signal from the overlying skeletal muscle. Considering all eight subjects, the metabolite concentrations were determined as: γ-ATP = 2.44 ± 0.16 (mean ± sd) mmol/l of wet tissue volume, α-ATP = 3.2 ± 0.63 mmol/l, β-ATP = 2.98 ± 0.45 mmol/l, Pi = 1.87 ± 0.25 mmol/l, PDE = 10.62 ± 2.20 mmol/l, and PME = 2.12 ± 0.51 mmol/l.

## Discussions

This study demonstrates the robust and effective combination of surface-spoiling inhomogeneous gradient and surface RF coil for detecting localized ^31^P spectra from human liver and thereby determining absolute phosphorus metabolite concentrations. The surface-spoiling gradient coil eliminates signal from the surface muscles while preserving the signal from deep-lying liver tissue. The signal-to-noise and linewidths of the resonances from the liver are preserved. The so determined quantitative phosphorus metabolite concentrations are in good agreement with previous reports employing other approaches (Table 1) [13, 15, 31–35]. In short, this method provides a straight-forward, robust, time-efficient approach to eliminate signal from surface tissue and quantify liver phosphorus metabolites in human studies.

**Table 1:** Calculated absolute metabolite results from previous studies in mmol.

	PME	Pi	PDE	γ-ATP	α-ATP	β-ATP
This study	2.12±0.51	1.87±0.25	10.62±2.2	2.44±0.21	3.2±0.63	2.98±0.45
Laufs et al. 2014	1.98±0.58	1.99±0.51	8.01±2.17	2.74±0.55	2.66±1.19	2.15±1.18
Noren et al. 2005	1.32±0.47	1.77±0.32	7.89±3.52	-	-	3.29±0.32
Corbin et al. 2004	3.69±0.56	1.18±0.07	7.64±0.7	-	-	3.2±0.15
Tosner et al. 2001	2.8±1.3	1.7±0.7	9.9±2.7	-	-	3.6±0.9
Sijens et al. 1998	2.52±0.37	1.38±0.27	12.5±1.98	-	-	2.5±0.33
Li et al. 1996	2.4±0.41	2.8±0.5	7.6±1.76	-	-	3.8±0.3
Oberhaensli et al. 1990	1.1±0.17	1.8±0.25	4.5±0.25	-	-	-

An important assumption for absolute quantification is that the volume of excitation is the same for phantom and *in vivo* experiments (i.e., equal contributing volumes for reference solution and liver tissue). The spoiling gradient field is similar for phantom and *in vivo* studies and decreases dramatically with increasing distance from the coil. However, for deeper tissue the different T_2_ of metabolite and phantom resonances can lead to differential dephasing and this effect can become significant at large TE. To minimize this effect, the gradient pulse duration was kept short (1 ms). As noted in a previous report, for T_2_ values within the range observed for *in vivo* phosphorus metabolite ^31^P resonances, this effect is small when the gradient duration is short (< 2 ms) and, thus, phosphorus metabolite ^31^P resonances with different T_2_ values are spoiled equally to within 4% [26]. For longer gradient durations these differences may become significant and should be taken into account. The gradient duration can be further reduced but would require larger driving current for similar spoiling depth.

We used 10 cm surface coil and it is conceivable that the presence of structures other than liver, such as stomach, might lead to contamination of the liver spectrum. Since stomach is a muscle tissue, a lack of PCr signal in the spectrum indicates that its contribution is negligible. Another issue that can impact the quantification is the presence of lung tissue in the sensitive volume of the coil since the air in the lungs would not contribute to the signal. This will make the volume of excitation different for *in vivo* and phantom experiments since the corresponding region in phantom experiment will contain the reference solution. To avoid this possibility, the RF coil was positioned carefully *via* the ^1^H scout images using the fiducial attached to the center of the coil as reference. In addition, since the sensitive volume of the surface coil is approximately a hemisphere defined by the coil radius, the effective diameter of the excitation volume is significantly smaller than the diameter of the coil. Thus, the position of lung and stomach were in regions far from the sensitive region of the surface coil.

A TR of 3 s was used in this study, which ensures that the Pi and ATP resonances are fully relaxed and no correction for partial saturation is needed. However, the T_1_ relaxation times of PME and PDE are long and large variations in these relaxation times have been reported among subjects [30]. Thus, the derived concentrations of PME and PDE may contain errors due to uncertainties in their ^31^P T_1_ relaxation times. The errors in determining concentrations of PME and PDE can be reduced by measuring their ^31^P T_1_ relaxation times as part of the overall quantification process. An additional MRS scan with different TR could be used to calculate the T_1_ of PME and PDE with modest addition to the total experiment time.

The gradient spoiling approach offers several advantages. (i) The efficiency of localization does not depend on the static magnetic field strength and same gradient coil can be used at any field strength without re-calibration. (ii) This approach is rather insensitive to motion since the gradient coil will move with the breathing motion when placed on the subject’s chest or abdomen. Because the spoiling depth is relative to the surface of the coil, it would, thus, not be affected by the breathing motion. (iii) The localization efficiency is not dependent on the chemical shift range of the nuclei studied but does depend on the gyromagnetic ratio of the spin system under study. In contrast, techniques using standard pulsed gradients in combination with frequency selective pulses are limited by the frequency range of metabolite resonances. This becomes more severe at high field strengths due to large chemical shift frequency dispersion, which can cause registration artifacts [36].

A disadvantage of the surface-spoiling surface-coil approach is lack of spatial resolution. However, the liver is a homogeneous organ and this technique can be useful for diffuse liver diseases such as non-alcoholic fatty liver disease, cirrhosis, and diabetes mellitus. Also, the short acquisition time makes it attractive for large-scale studies. The approach presented is not limited to pulse-and-acquire sequences but can be used in conjunction with other pulse sequences to improve the localization efficiency. For example, this approach has been previously employed with a GRE pulse sequence to reduce the field of view in cardiac MRI studies [23]. It is possible to combine this approach with CSI to image deeper lying organs such as heart, kidney and pancreas.

The gradient spoiling approach described here requires external hardware, which can introduce noise. A low pass filter eliminates such concerns. The currents used in this study were fairly low and the gradient field dropped off quickly with distance from the coil. Eddy current distortions and significant spectral resonance broadening from the targeted tissue were not observed. All human studies reported herein were performed with free breathing, which can lead to spectral distortion and line broadening. Respiratory gated studies with localized shimming can further improve the spectral quality.

In summary, the surface-spoiling gradient coil approach provides a simple and efficient method to localize MRS signals from targeted deep-lying organs. Feasibility was demonstrated with human subjects using a simple pulse-and-acquire sequence and, which, as a consequence, permits short measurement time. Hence, this method can be utilized in large-scale clinical and research studies. The method also enables quantification of molar concentrations of phosphorus metabolites in liver and the results were found to be consistent with data from previous reports [11–16]. Since the approach is independent of magnetic field strength, it can be used in high-field studies where chemical shift artifacts or SAR can challenge other pulse sequences.

## Supporting Information

S1 File Tables listing all the computed value. Data for two compartment phantom demonstrating the suppression of signal from the surface compartment as the current through the gradient coil is increased Fig 4 (Table A). Data from single compartment phantom used to calibrate RF pulses in absence and presence of spoiling gradient field Fig 5 (Table B). In vivo spectra demonstrating suppression of signal arising from surface tissue using meanderline gradient coil Fig 6 (Table C). 1D profiles from the phantom in absence and presence of spoiling gradient showing region of signal suppression Fig 7 (Table D). Data representing the off-resonance efficiency of the adiabatic excitation pulse Fig 8 (Table E). Amplitude of all resonances from all studies (Table F).
(XLSX)

S1 Script Function to calculate the surface-spoiling magnetic field Bz distribution along the Z-axis for meanderline spoiling gradient coil. The field can be calculated for different spacing between meanderline current elements, varying input current and different depths from the coil surface. The Biot-Savart equation was used in these calculations.
(M) Click here for additional data file.
